# Successful Delayed Elective Separation of Pygopagus Conjoined Twins With Shared Spinal Cord and Dura in a Resource‐Limited Setting: A Case Report

**DOI:** 10.1002/ccr3.73657

**Published:** 2026-10-02

**Authors:** Z. U. M. Wahid, Saiful Islam Chowdhury, Nushrat Islam, Sumaya Khan Mifty

**Affiliations:** ^1^ Dhaka Medical College and Hospital Dhaka Bangladesh; ^2^ Shaheed Suhrawardy Medical College and Hospital Dhaka Bangladesh

**Keywords:** delayed separation, dural fusion, microsurgical technique, pygopagus conjoined twins, spinal cord sharing

## Abstract

Conjoined twins are a rare consequence of aberrant monozygotic twinning; one of the varieties is pygopagus twins, which are fused at the sacral region and may involve complex neural and dural sharing. We report a case of female pygopagus conjoined twins with shared spinal cord and dural sharing who underwent successful delayed elective separation at 6 months of age in a resource‐limited tertiary care setting. Initial imaging revealed sacral deficiency, dural fusion, and spinal cord involvement without bony fusion. Surgery was postponed to allow nutritional optimization and adequate tissue growth. Nutritional readiness and local tissue adequacy were assessed through longitudinal clinical evaluation of weight‐gain velocity, visceral protein sufficiency, and dermal mobility relative to pelvic landmarks, without relying on mechanical tissue expanders. Under general anesthesia, meticulous microsurgical separation of the fused dura and neural elements was performed, followed by watertight dural closure. Separation was accomplished without intraoperative neurophysiological monitoring, relying entirely on direct magnification and stepwise dissection. The postoperative course was largely uneventful except for transient pneumonia in one twin. Both infants were discharged on postoperative Day 12 in stable condition with intact neurological function. Delayed planned separation with multidisciplinary coordination and meticulous surgical technique can result in favorable outcomes, even in resource‐constrained settings lacking advanced neurophysiological mapping facilities.

## Key Clinical Message

Pygopagus conjoined twins with shared spinal cord and dura present a rare surgical challenge requiring careful anatomical assessment and planning. Delayed elective separation allows optimization of nutritional status and tissue development, improving operative safety and outcomes. Even in resource‐constrained tertiary environments where advanced intraoperative neurophysiological mapping is inaccessible, disciplined microscopic dissection, adherence to fixed osseous‐dural landmarks, and stepwise neural preservation can achieve favorable functional outcomes with intact motor and visceral sphincteric control.

## 1 Introduction

Conjoined twins, popularly known as Siamese twins, are suggested to result from aberrant embryogenesis. Conjoined twins are classified based on their site of attachment. The main types are omphalopagus (abdomen), thoracopagus (thorax), cephalopagus (ventrally—head to umbilicus), ischiopagus (pelvis), parapagus (laterally—body side), craniopagus (head), pygopagus (sacrum), and rachipagus (vertebral column) [1, 2].

The pygopagus variety is a type in which twins are joined at the rump and have a union of the gastrointestinal and genitourinary systems as well as occasional involvement of neural elements [3].

Conjoined twins share some organs that are vital for survival, like the heart; these twins are almost impossible to save, but there are some cases wherein there is evidence of their survival [4].

Imaging plays an essential role in the diagnosis and follow‐up of conjoined twins. While ultrasound is often the screening modality of choice, CT scan and MRI are also carried out for better anatomical delineation and further characterization as and when necessary [5].

Planned elective separation between 4 and 12 months of age is considered optimal, allowing adequate somatic growth, tissue expansion, and detailed imaging for surgical planning. We present a successful delayed elective separation of female pygopagus twins with shared spinal cord and dura performed in a resource‐limited tertiary care center.

## 2 Case History

A 2‐day‐old female conjoined twin pair came to a tertiary medical hospital with the diagnosis of conjoined twin pygopagus variety. During history taking, mother's antenatal period was uneventful. She had several antenatal checkups, and during a USG on 20 weeks it came to light that she was going to give birth to conjoined twins. After that, she was in constant contact with her gynecologist. Her remaining antenatal period was uneventful. She also couldn't mention any similar incident of this sort in her family. She had a preplanned LUCS in a government medical hospital. The children's delivery was uneventful, and her postpartum period was also nothing exceptional. After delivery, she was referred to a tertiary medical college for separation of the babies.

### 2.1 Diagnostic Evaluation & Baseline Investigations

The two children were diagnosed as “Twin A” and “Twin B”. Both children were born with a birth weight of 3.6 kg together. Both children were fused in the lower part of the body in the sacral region with soft tissue bridging (Figure 1). The length of the area of joining the skin from the outside is about 10 cm in length.

**FIGURE 1:**
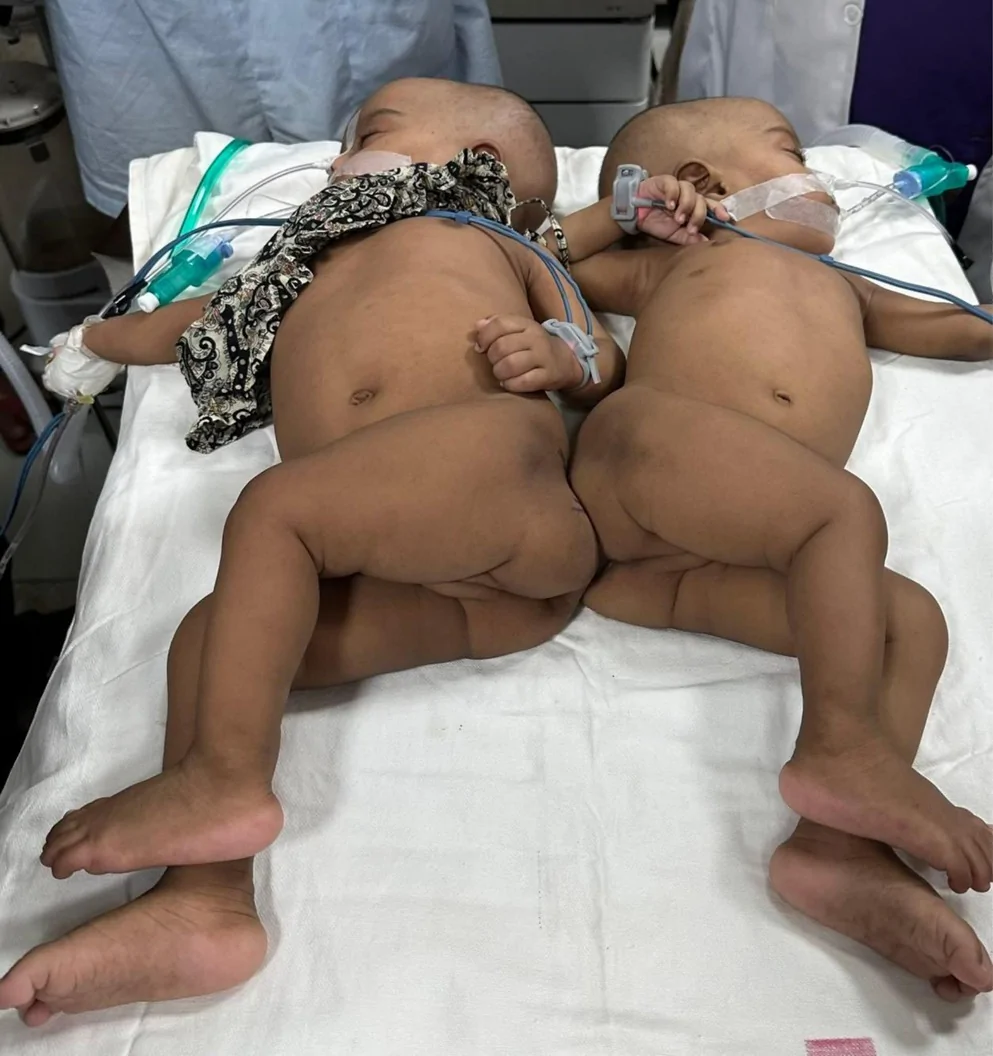
Conjoined Twin (Pygopagus Variety‐ Twins fused in the lower part of the body in the sacral region with soft tissue bridging).

Baseline investigations at admission demonstrated stable physiological parameters across both infants (Table 1).

**TABLE 1:** Initial routine investigations of the twins.

Test name	Twin A	Twin B	Reference value
CBC	Hb: 14.8 RBC: 5.54 million/mm^3^ Platelet: 341,000 WBC: 16.01 k Neutrophil‐33.7% Leukocyte‐52.8% Monocyte‐ 6.5% Eosinophil‐7.0% Basophil‐0.1%	Hb: 14.3 RBC: 5.49 million/mm^3^ Platelet: 368,000 WBC: 12.29 k Neutrophil‐43.6% Leukocyte‐38.6% Monocyte‐9.4% Eosinophil‐8.4% Basophil‐0.2%	Hb: 13–18 (male) 11.5–15.5 (female) RBC: 4.5–5.5 Platelet: 150000–450,000 WBC: 4000–11,000 Neutrophil‐40%–70% Leukocyte‐20%–45% Monocyte‐02%–08% Eosinophil‐01%–05% Basophil‐0%–01%
Serum creatinine (mg/dl)	0.72	0.62	Male: 0.6–1.5 Female: 0.4–1.2 Child: 0.2–0.7
Total serum bilirubin (mg/dl)	7.1	7.0	0.3–1.2
Serum electrolytes (mmol/l)	Sodium‐139.0 Potassium‐5.7 Chlorine‐106.7	Sodium‐128.0 Potassium‐5.32 Chlorine‐96.2	Sodium‐135–145 Potassium‐3.5–5.5 Chlorine‐95–105
Serum albumin (gm/dl)	4.3	3.7	Adult: 3.4–5.00 Neonate: 2.6–4.4
Echocardiography	Situs Solitus, levocardia Good biventricular function No shunt anomaly Normal PA pressure (PASP = 20 mmHg)	Situs Solitus, levocardia Good biventricular function No shunt anomaly Normal PA pressure (PASP = 20 mmHg)	
USG of Whole Abdomen	No significant abnormality is detected	No significant abnormality is detected	

### 2.2 Preoperative Optimization: Nutritional Assessment, Goals & Tissue Strategy

A multidisciplinary hospital board was convened, comprising pediatric surgeons, neurosurgeons, pediatricians, anesthesiologists, and a dedicated pediatric clinical nutritionist. Initial assessment determined that the infants were clinically underweight (combined birth weight of 3.6 kg) to withstand the physiological trauma of prolonged separation surgery. Furthermore, the available skin and subcutaneous tissue around the conjoined pelvic junction were deemed insufficient for tension‐free primary coverage. The board unanimously decided to postpone elective intervention and establish a structured nutritional rehabilitation protocol.

**Longitudinal Nutritional Goals and Physiological Landmarks:** Surgical readiness was evaluated through continuous physiological and anthropometric monitoring rather than an arbitrary numerical threshold or rigid protocol score. A clinical nutritionist supervised maternal breastfeeding and instituted calorie‐dense nutritional supplementation:

- **Somatic Weight Gain Velocity:** The primary physiological target was a consistent weight gain velocity of 20 to 30 g/day per infant, targeting a doubling of baseline weight before intervention. Over the 6 month delay, the twins achieved a combined body weight of 8.9 kg, crossing into safe pediatric weight‐for‐age parameters.
- **Visceral Protein Stores and Hematological Stability:** Nutritional adequacy was corroborated biochemically by maintaining serum albumin levels above 3.5 g/dL (Twin A: 4.3 g/dL, Twin B: 3.7 g/dL) and sustaining normal electrolyte and hemoglobin indices. These markers confirmed sufficient physiological reserves to tolerate intraoperative blood loss, lengthy anesthesia, and post‐separation hypercatabolism.
- **Assessment of Tissue Coverage & Decision Regarding Tissue Expanders:** The adequacy of regional soft tissue for tension‐free surgical closure was assessed dynamically using regional biomechanical landmarks.
- **Biological Tissue Creep and Elasticity:** Serial physical examinations evaluated the pinch‐laxity and dermal mobility of the lateral gluteal and flank integument relative to fixed skeletal landmarks—specifically the sacrococcygeal junction, the posterior superior iliac spines (PSIS), and the anal margins. Natural somatic growth over 6 months induced spontaneous mechanical tissue creep, generating redundant lateral skin folds.
- **Microvascular Perfusion:** Skin vascularity was verified through robust capillary refill (< 2 s) and the absence of scar tethering or dermal attenuation across the junction.
- **Consideration of Tissue Expanders:** The multidisciplinary team formally considered the insertion of mechanical tissue expanders. However, their use was decided against due to: (1) stringent institutional resource limitations, (2) heightened risks of expander erosion, hematoma, and catastrophic implant infection given the proximity to the diaper/perineal region, and (3) clinical evidence that spontaneous somatic growth and tissue relaxation over the 6 month delay yielded sufficient cutaneous redundancy to allow primary local flap reconstruction without synthetic prostheses.

### 2.3 Neuroimaging Findings

After 3 months, initial imagings were done to check the complex structure of the children's conjoined portions. Initial CT scan with 3D Figure 2 reconstruction revealed normal spinal anatomy. Then MRI of the pelvis Figure 3 was done, with the findings of: the sacrum is deficient in both fetuses, there is fusion of both fetuses in the lower posterior part by soft tissue bridging; the spinal cord seems to be fused together; the hip bones in both fetuses are developing.

**FIGURE 2:**
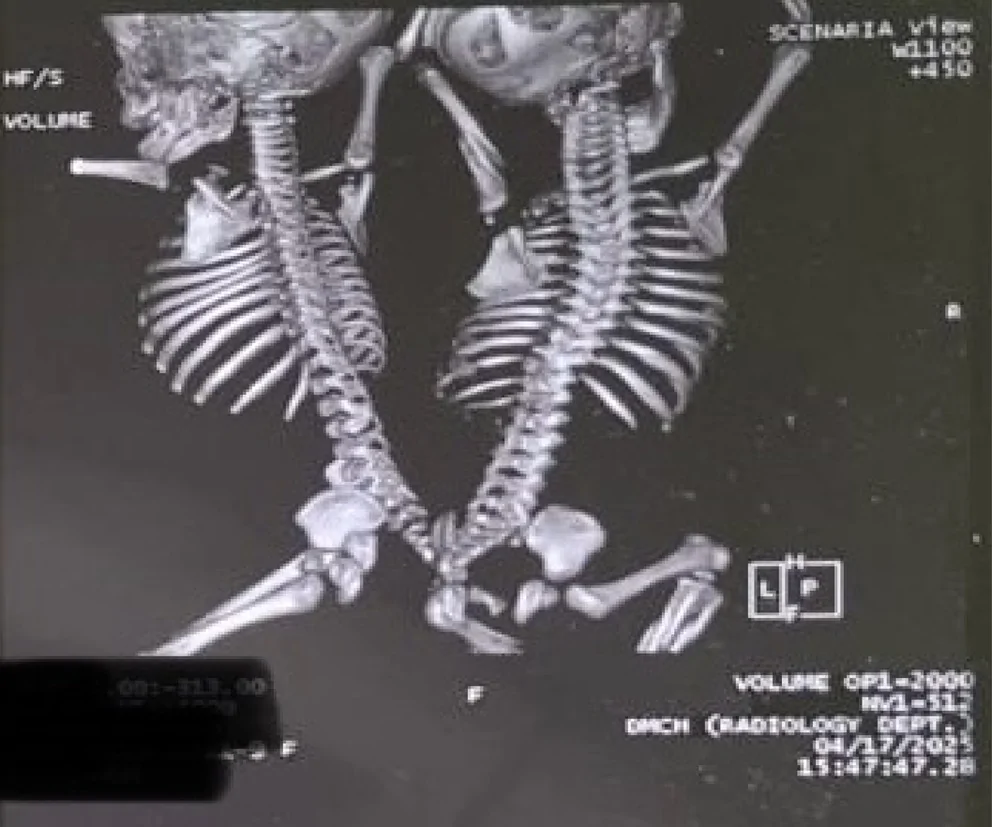
Representative posterior three‐dimensional (3D) computed tomography (CT) skeletal reconstruction of the pygopagus conjoined twins at 3 months of age. The volume‐rendered view demonstrates independent, anatomically normal cervical, thoracic, and lumbar spines with preserved vertebral alignment and intervertebral disc spaces. There is no osseous bridging or pelvic bone fusion; skeletal contact is limited to the lower sacrococcygeal region within the soft‐tissue bridge.

**FIGURE 3:**
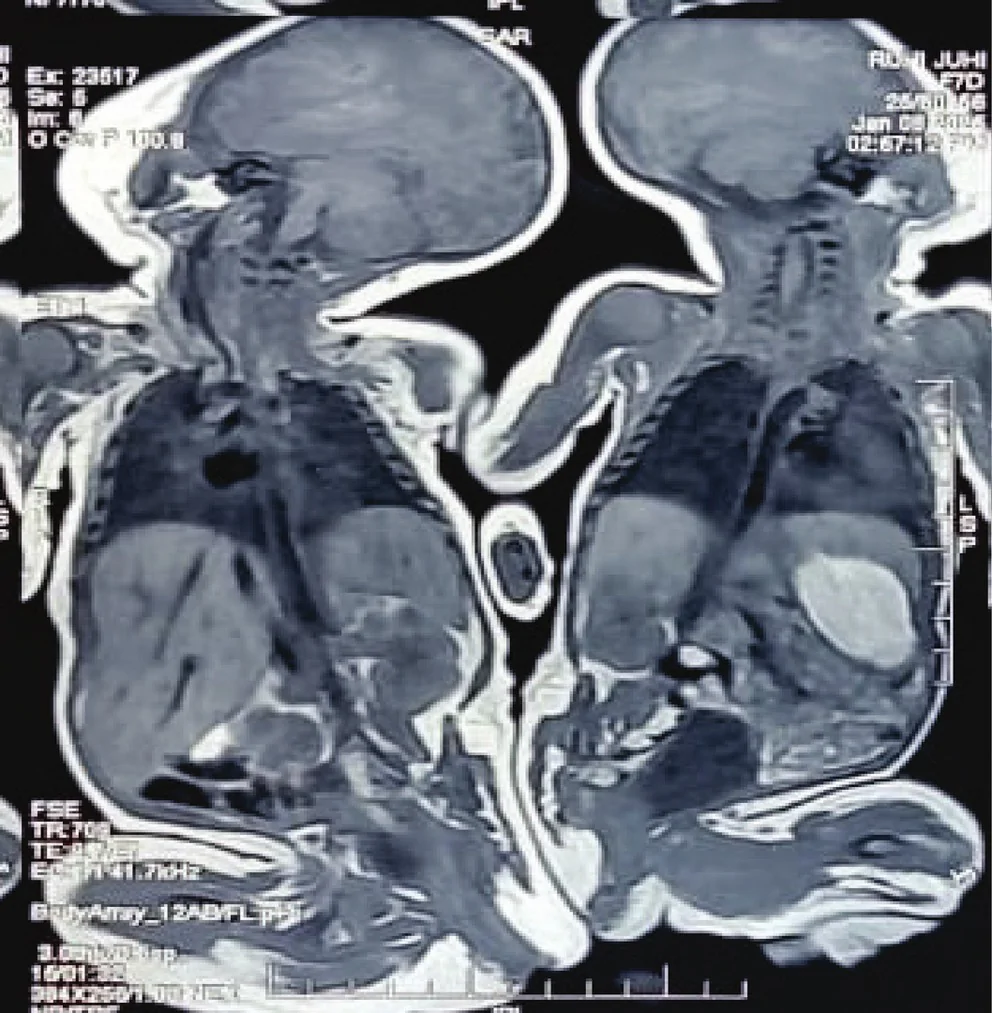
Representative coronal T1‐weighted pelvic and trunk magnetic resonance imaging (MRI) of the pygopagus conjoined twins at 3 months of age. The scan reveals independent thoracic and abdominal visceral compartments without cross‐visceral organ sharing. Posterior fusion is confirmed across the sacrococcygeal region via a common soft‐tissue bridge. The lower spinal axes converge caudally toward the junction, showing sacral deficiency and conjoined distal dural and spinal cord elements terminating near the sacrococcygeal level, while the developing pelvic structures and external perineal anatomy remain distinct.

After reviewing all the reports, the board of doctors decided that they could proceed for operation after 1 month. But Twin A was diagnosed with pneumonia, and the surgery was postponed, and 1 month later, the surgery took place.

### 2.4 Surgical Separation Technique

**Intraoperative Decision‐Making Without Neuromonitoring:** Advanced intraoperative neurophysiological monitoring (IONM)—such as continuous somatosensory evoked potentials (SSEPs), transcranial motor evoked potentials (MEPs), and electromyographic root stimulation—was unavailable due to resource limitations. Consequently, intraoperative neural preservation depended entirely on high‐magnification operating microscopy, meticulous microsurgical technique, and disciplined adherence to fixed anatomical landmarks.

Under general endotracheal anesthesia, the infants were positioned in a synchronized lateral decubitus position. A modified curvilinear zigzag (interlocking S‐pattern) incision was designed over the posterior soft‐tissue bridge. This geometric flap design was chosen to distribute skin tension evenly, prevent straight‐line scar contracture over the sacrum, and optimize cutaneous flap interdigitation for each twin post‐separation.

### 2.5 Dissection and Neural Separation Steps

- **Anatomical Landmark Identification:** Subcutaneous flaps were elevated with bipolar electrocautery down to the paraspinal muscular fascia. The sacrococcygeal junction was identified and utilized as the principal surgical landmark to anchor the exposure.
- **Dural Opening:** The thecal envelope was confirmed to be fused at its distal pole near the sacrococcygeal boundary. Under high‐power microscopic visualization, the shared dura was carefully incised longitudinally along the midline raphe.
- **Nerve Root Tracing:** Inside the common thecal chamber, the nerve roots for each twin were clearly identified. Individual nerve roots were systematically traced proximally toward their cord origin and distally toward their corresponding neural foramina. This bilateral visual confirmation assured the surgical team that each root belonged unequivocally to its respective infant before any neural division.
- **Cord Division & Dural Envelope Separation:** With gentle blunt micro‐dissection and continuous saline irrigation, the conjoined lower spinal cord elements were carefully separated without sacrificing functioning neural tissue of either twin. The thecal sac was then cleanly partitioned into two distinct envelopes.

After detaching both babies, they were shifted to another table and the operation was resumed. The thecal sac was repaired under microscope with 6.0 Prolene RB, and dura was closed in a water tight fashion. After the fascial layer was strengthened, CSF leakage was checked, and there was none after valsalva maneuver. The wound was closed in layers, and skin suture was given (Figure 4).

**FIGURE 4:**
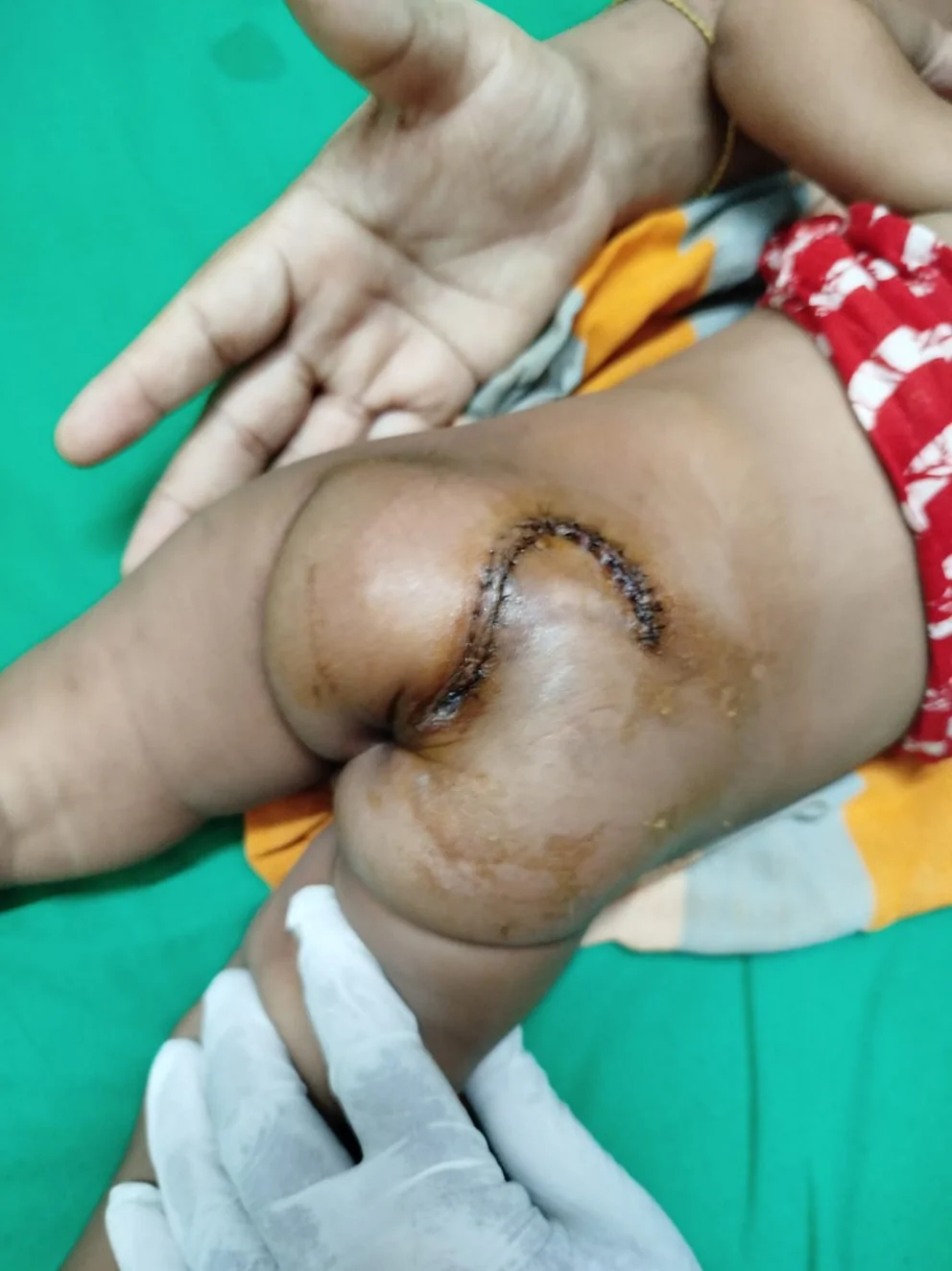
Postoperative clinical photograph of the separated infant prior to discharge. The image demonstrates primary, tension‐free closure across the sacrococcygeal and gluteal region using a modified curvilinear zigzag flap design. The surgical wound is well‐apposed with intact sutures, showing viable local skin margins and no evidence of wound dehiscence, flap necrosis, infection, or cerebrospinal fluid (CSF) leakage. Preserved perineal architecture and anal contour are maintained.

## 3 Outcome and Follow‐Up

Post‐operative period was uneventful without surgical site infections, except on the 3rd day, when Twin B developed pneumonia and was managed accordingly. On the 12th post‐operative day, they were given release with basic vitamin supplements, and both of the babies are in healthy condition and leading an independent life.

### 3.1 Clinical Neurological and Functional Examination

**Detailed clinical neurological examinations were conducted before hospital discharge:**

- **Lower Limb Motor Function:** Both infants exhibited active, symmetric, and spontaneous movements of the bilateral lower extremities against gravity (kick cycles, foot flexion/extension). Muscle tone across the hip flexors, quadriceps, and gastrocnemius muscles was normal and symmetric. Deep tendon reflexes (patellar and Achilles) were brisk and bilaterally equal (+2/4).
- **Sensory Response:** Intact and symmetric sensory withdrawal responses were observed across all lumbar and sacral dermatomes (L1–S3) upon gentle tactile stimulation.
- **Sphincter Function:** Both twins exhibited normal, robust anal wink reflexes, confirming preservation of the S2–S4 sacral autonomic and pudendal pathways. Both infants demonstrated normal spontaneous fecal evacuation and independent voluntary voiding patterns without signs of urinary retention or overflow dribbling.

## 4 Discussion

### 4.1 Epidemiology

Conjoined twins are a rare form of monozygotic and monochorionic twinning in which babies are physically connected to each other. The incidence is 1 in 100,000 to 250,000 [6, 7]. Approximately 184 successful separations have been described, and a multidisciplinary approach is required for the surgical management [1]. But approximately half of the cases result in stillbirth; the actual incidence is much higher, and the incidence is higher in females than males in a ratio of 3:1 [7]. Our report highlights a case of female conjoined twins. Conjoined twins are classified according to the site of fusion, and there are 15 recognized types, among which more than 70% of cases represents five types (Thoracopagus, Omphalopagus, Pygopagus, Ischiopagus, Craniopagus). The most frequent form is thoraco‐omphalopagus, comprising nearly 28% of reported cases [8]. On the other hand, pygopagus twins are rarer than others, comprising 6% to 19% [7] of total cases and here in our case we will discuss the successful separation of Pygopagus conjoined twins with involvement of the spine [9].

### 4.2 Management

The management of conjoined twins can be divided into three categories. Non‐operative management, Emergency separation and Planned separation [10]. The preferred management is planned elective separation, and the optimal age of it is 4–12 months of age, allowing sufficient time for somatic growth, tissue expansion, and comprehensive imaging, which enables precise delineation of the anatomical fusion and associated anomalies and facilitates optimal surgical planning [11, 12]. In our cases, the twins delivered by Cesarean section came to a tertiary medical college where the conjoined twins diagnosis was made by MRI and CT Scan. MRI of the pelvis shows the type of conjoined twins is Pygopagus variety with spinal cord involvement, which is rare among cases of pygopagus. Of these, surgical separations were performed in 26 cases; dural sharing was present in 17 cases, while spinal cord sharing was present in 10 cases [13].

In our case, both spinal cords were joined together, and dural sharing was also present. After the diagnosis, a multidisciplinary board was formed in order to separate the twins, but the children were grossly underweight to undergo the lengthy and complex procedure, and there was also not enough tissue around the conjoined region to separate them and go for reconstruction. For this reason, the board decided to delay the operation for 3 months to improve their nutrition. After 3 months, one of the twins developed pneumonia, which delayed the operation for 3 more months and finally the board decided to perform the surgery 6 months from the date of birth of the twins, which aligns with the optimal age for planned elective separation.

In case of spinal cord involvement in the pygopagus variety of conjoined twins, detailed neurological mapping is necessary in order to plan the successful separation of the twins; otherwise complications may arise [14]. In our cases, detailed neurological mapping couldn't be done due to limited resources of the medical system of a developing country, and they had to be more careful to avoid neurological injuries while relying mostly on anatomical landmarks, careful nerve root separation and stepwise separation. Under General Anesthesia, the operation was started and after incision of the dura, nerve roots were separated, and thecal sac was incised to separate the twins. During this kind of operation where spinal cords are joined and the dural sac is involved, CSF leakage is a common complication [13], but in our case, surgeons smartly handled the situation and controlled CSF leakage through watertight closure.

However, the surgical procedure for conjoined twins is complicated; the main challenge lies after the operation. The survival rate of conjoined twins after operation is 16.7%–50%, depending on the type of operation and whether it is elective or emergency [15]. But the fatality rate becomes higher when there is a neurological association between the twins; especially, it is difficult for one of the twins to survive independently while separating the joined spinal cord [16]. In our case, both of the twins survived after the operation, and both of them are doing well independently, which is a great success for the surgeons.

**Microsurgical Nuances in Resource‐Limited Settings:** In well‐resourced centers, intraoperative neurophysiological mapping (MEPs, SSEPs, pudendal nerve stimulation) is considered the gold standard for separating shared thecal and cord tissue [13, 14]. When these technologies are unavailable, surgeons must not compromise precision. In this case, success was achieved through three vital surgical strategies:

- **Fixed Skeletal Anchoring:** Utilizing the sacrococcygeal junction as the principal landmark reliably established the surgical entry level.
- **Stepwise Magnified Micro‐dissection:** High‐power microscopic visualization permitted clear discrimination between fascial boundaries and neural envelopes.
- **Individual Rootlet Verification:** Tracking each nerve bundle to its definitive pelvic exit before dural division ensured that no functional neural tissue belonging to either infant was compromised.

Furthermore, watertight 6–0 polypropylene dural closure backed by autologous fascial reinforcement prevented CSF leakage—a historically frequent and lethal complication in pygopagus separations [13].

### 4.3 Study Limitations

This report has several limitations that must be acknowledged:

- **Nutritional & Tissue Assessment:** Nutritional status and skin availability were evaluated based on clinical expertise, growth velocity, and visceral biochemical parameters rather than a validated pediatric anthropometric scoring tool or objective skin perfusion device.
- **Lack of Intraoperative Electrophysiology:** While anatomical preservation was verified by direct microsurgical inspection, the absence of intraoperative neurophysiological monitoring precluded real‐time functional electrophysiological feedback during cord division.
- **Follow‐Up Diagnostics:** Neurological and sphincter assessments were based on comprehensive clinical examinations. Advanced urodynamic studies and electrodiagnostic electromyography could not be performed due to local facility constraints.

## 5 Conclusion

Pygopagus conjoined twins with spinal cord and dural sharing present exceptional surgical challenges and are associated with high morbidity and mortality. This case demonstrates that successful separation is possible through careful multidisciplinary planning, delayed elective surgery for nutritional optimization, and meticulous surgical technique, even in the absence of advanced neurophysiological mapping. The favorable survival and functional outcome of both twins highlight the feasibility of managing complex conjoined twin separations in resource‐limited settings.

## Author Contributions

**Z. U. M. Wahid:** conceptualization, methodology, validation, visualization, writing – original draft, project administration, resources. **Saiful Islam Chowdhury:** conceptualization, writing – original draft, methodology, validation, visualization, project administration, resources. **Nushrat Islam:** conceptualization, validation, writing – review and editing. **Sumaya Khan Mifty:** conceptualization, validation, writing – review and editing.

## Funding

The authors have nothing to report.

## Disclosure

Declaration of AI‐Assisted Tool Use: In accordance with Wiley's publishing ethics and policy on artificial intelligence, we declare that generative AI assistance (Gemini, Google) was utilized during manuscript preparation strictly for language editing, stylistic refinement, and structuring the response letter. No original clinical findings or radiographic analyses were generated or altered by AI tools, and all authors have reviewed, verified, and approved the final submission.

## Ethics Statement

The Institutional Review Board reviewed the case and granted an exemption from formal ethical approval, as this report involves a single anonymized patient case.

## Consent

Written informed consent was obtained from the patient to publish this report in accordance with the journal's patient consent policy.

## Conflicts of Interest

The authors declare no conflicts of interest.

## Data Availability

The data that support the findings of this study are available from the corresponding author upon reasonable request.
